# Sensors for Fetal Hypoxia and Metabolic Acidosis: A Review

**DOI:** 10.3390/s18082648

**Published:** 2018-08-13

**Authors:** Gerard Cummins, Jessica Kremer, Anne Bernassau, Andrew Brown, Helen L. Bridle, Holger Schulze, Till T. Bachmann, Michael Crichton, Fiona C. Denison, Marc P. Y. Desmulliez

**Affiliations:** 1 Institute of Sensors, Signals and Systems, Heriot-Watt University, Riccarton EH14 4AS, Scotland, UK; kremer-jessica@gmx.de (J.K.); A.Bernassau@hw.ac.uk (A.B.); H.L.Bridle@hw.ac.uk (H.L.B.); M.Desmulliez@hw.ac.uk (M.P.Y.D.); 2 MRC Centre for Reproductive Health, Queen’s Medical Research Institute, University of Edinburgh, Edinburgh EH16 4TJ, Scotland, UK; brown.andrewpatrick@gmail.com (A.B.); fiona.denison@ed.ac.uk (F.C.D.); 3 Division of Infection and Pathway Medicine, Edinburgh Medical School, The Chancellor’s Building, The University of Edinburgh, Edinburgh EH16 4SB, Scotland, UK; Holger.Schulze@ed.ac.uk (H.S.); till.bachmann@ed.ac.uk (T.T.B.); 4 Institute of Mechanical, Processing and Energy Engineering, Heriot-Watt University, Riccarton EH14 4AS, Scotland, UK; m.crichton@hw.ac.uk

**Keywords:** lactate, electrochemical sensing, optical sensing, lactate oxidase, lactate hydrogenase, fetal monitoring, hypoxia, obstetrics, fetal blood sampling

## Abstract

This article reviews existing clinical practices and sensor research undertaken to monitor fetal well-being during labour. Current clinical practices that include fetal heart rate monitoring and fetal scalp blood sampling are shown to be either inadequate or time-consuming. Monitoring of lactate in blood is identified as a potential alternative for intrapartum fetal monitoring due to its ability to distinguish between different types of acidosis. A literature review from a medical and technical perspective is presented to identify the current advancements in the field of lactate sensors for this application. It is concluded that a less invasive and a more continuous monitoring device is required to fulfill the clinical needs of intrapartum fetal monitoring. Potential specifications for such a system are also presented in this paper.

## 1. Introduction and Clinical Motivation

Fetal monitoring during labor is routinely used in high and middle-income countries to detect fetuses at risk of hypoxia, acidosis, and associated sequelae including hypoxic-ischemic encephalopathy, cerebral palsy, and death [1]. Such monitoring involves intermittent or continuous fetal heart rate monitoring with external ultrasound transducers applied to the maternal abdominal wall. Continuous cardiotocography (CTG), which was invented in the 1960s [2,3], involves simultaneous assessment of the fetal heart rate and maternal uterine activity. It is recommended for intrapartum monitoring in women at high risk of complications [1,4]. While this technique is non-invasive, it has a low positive and negative predictive value of 30% and 86%, respectively, for the detection of fetal hypoxia and cerebral palsy [5,6]. This technique, coupled with the high intra-observer variability in its assessment [7], can lead to unnecessary intervention including a caesarean section [8]. Therefore, CTG is best considered a screening tool for identifying fetuses at risk, which enables clinicians to further investigate or treat the condition.

Monitoring of the fetal heart rate through the maternal abdominal wall can sometimes be difficult. For example, maternal adiposity, the position of the fetus, or twin pregnancy could all pose complications for monitoring the fetal heart rate. A fetal scalp electrode (FSE) can be applied directly to the baby’s head to measure heart rate or fetal electrocardiogram (ECG). Although the FSE usually provides a more stable fetal heart rate signal, there is a higher risk of injury and infection due to the device needing to puncture the fetal scalp to obtain a signal [9]. The positive predictive value for detecting fetal hypoxia and cerebral palsy is also no different from that obtained through external ultrasound transducers.

In an attempt to improve the detection of the fetal compromise, alternative screening methods have been proposed either as an isolated screening test or in combination with other screening tests such as CTG. Fetal oxygenation has been measured through a device attached to the clinician’s finger [10,11] or by directly using a pulse oximetry device embedded into the hook of the FSE [12]. However, although it is possible to measure fetal oxygenation via the fetal scalp, the addition of fetal pulse oximetry neither reduces overall caesarean section rates nor improves clinical outcomes [13]. Fetal ECG has also been developed as a screening method for detecting the intrapartum fetal compromise. Fetal ECG signals are collected through an FSE with a reference electrode attached to the maternal thigh. Although initial randomized trials showed that the technology has the potential to reduce unnecessary operative intervention and neonatal metabolic acidosis [14,15], more recent studies have been equivocal [16,17] or have not demonstrated benefits [18]. 

Regardless of what screening test is used, a diagnostic test of fetal well-being is ultimately needed to inform decision-making. Several methods have been proposed either as an alternative or adjunct to CTG. Fetal scalp blood sampling (FBS) is the most commonly used since it allows direct measurement of biochemical parameters of acidosis such as pH, base deficit (BD), and lactate. FBS for pH measurement was first described by Saling in 1964 [19,20]. The sampling technique, which has changed little since then, requires cervical dilatation of 3 cm or more for adequate visualization of the fetal scalp using an amnioscope (a modified speculum), which is inserted vaginally. A small incision is made on the fetal scalp before collecting 30 to 50 µL of blood into a heparinized capillary tube. The blood is then analyzed in a blood gas analyzer maintained centrally in the labor ward [21]. This process is invasive, uncomfortable for women, and time-consuming. It takes between 12 to 25 minutes per sample [22]. Furthermore, it is associated with a failure rate of up to 20% due to technical and operator-related factors [22,23] such as insufficient sample volume, contamination with air or amniotic fluid [24], and calibration of the blood gas analyzer at the time of sampling [25]. In the past decade, lactate has been adopted as an alternative to pH measurand in FBS in Australia and much of Europe since it appears to be a better predictor of long-term neonatal outcomes. Furthermore, point-of-care meters have simplified the measurement processes [26,27].

Regardless of the measurand, FBS requires repeated samples to monitor changes in fetal physiology that can occur rapidly during labor. A continuous, real-time method of assessing the fetal acid-base status is, therefore, required to enable earlier identification and delivery of the hypoxic baby before irreversible brain damage occurs. This review explores recent technical advances in the field of sensors that could help meet the currently unmet clinical need for continuous fetal bio-sensing during labor.

## 2. Fetus-Mother Physiology

### 2.1. Metabolic Pathways During Blood Oxygenation and Deoxygenation 

**Figure 1:**
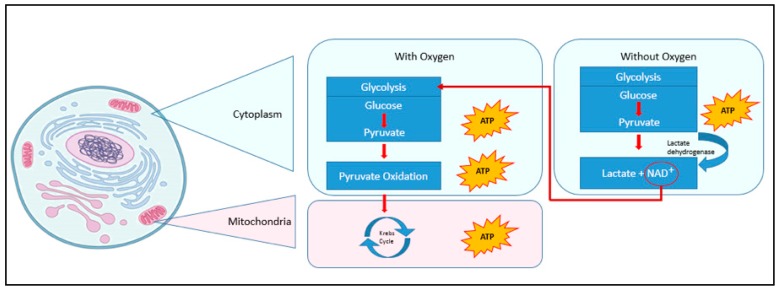
Aerobic and anaerobic production pathways of ATP.

Oxygen (O_2_) provides the basis for energy production from glucose [28]. The presence or absence of oxygen leads to different biochemical pathways for energy production, which is shown in Figure 1.

In the presence of O_2_, the human body generates energy in the form of adenosine triphosphate (ATP) by producing pyruvate from glucose (glycolysis), its oxidation, and the Krebs cycle. However, if the oxygen supply is limited, energy production is less efficient since the pyruvate is broken down into lactate nicotinamide adenine dinucleotide (NAD^+^) by lactate dehydrogenase in the presence of the reduced form of nicotinamide adenine dinucleotide (NADH) and hydrogen (H^+^) ions. NAD^+^ is vital for maintaining limited energy production through glycolysis (red arrow in Figure 1). The lactate is itself broken down into lactic acid and H^+^ ions, which causes the pH value to decrease [29]. When oxygen becomes available again, the process is reversed and lactate dehydrogenase converts lactate back into pyruvate, which is then used for more efficient energy production in the Krebs cycle [29]. There is, therefore, a strong correlation between the presence of oxygen and the production of lactate and H^+^ ions.

### 2.2. Physiological Effects of Lack of Oxygenation in the Fetus

During pregnancy, the provision of gas exchange (O_2_ and carbon dioxide (CO_2_)) is carried out in utero with the placenta replacing the function of the lungs of the fetus. Deoxygenated blood is pumped by the fetal heart through the two umbilical arteries to the placenta while oxygenated blood is pumped back into the fetal system through the single umbilical vein. During contractions, the perfusion in the placenta can be restricted, which leads to oxygen deficiency [29] and two different types of acidosis, which include respiratory and metabolic acidosis.

- *Respiratory acidosis* is due to the accumulation of carbon dioxide produced because of normal metabolism. Although the concentration of H^+^ ions increases, respiratory acidosis, in itself, is not associated with adverse neurological outcomes because the fetus implements compensatory methods [1,30].
- *Metabolic acidosis* occurs when the fetus receives inadequate oxygen to maintain normal metabolism, which forces a switch to anaerobic metabolism. This results in the formation of lactic acid and, when the buffering capacity of the tissues is exhausted, a decrease in pH. Since a lack of oxygen eventually leads to cell death, prolonged exposure to this situation can lead to postnatal neurological complications such as short-term hypoxic-ischemic encephalopathy or long-term disabilities such as spastic quadriplegia [28]. Profound intrapartum asphyxia can result in stillbirth [31] or neonatal death [4,32].

## 3. Measurands for the Detection of Fetal Hypoxia 

A range of parameters can be measured in fetal blood sampled during FBS to diagnose fetal acidosis and inform clinical management. 

### 3.1. pH

The normal arterial pH of a healthy fetus is about 7.35 and is regulated primarily by gas exchange at the placenta. There is a physiological decrease in pH during labor such that the mean umbilical artery pH at birth is 7.25 [1]. A further decrease in pH may occur as a result of either respiratory or metabolic acidosis [26,30].

Saling et al. suggested a threshold pH below 7.20 in order to intervene in his original work [33] and this was supported by later studies, which formed the basis for current international guidelines for interpreting fetal scalp pH (Table 1). However, large observational studies show that the association between low pH and neonatal neurological morbidity is weak in babies with an umbilical artery pH above 7.00 [34,35]. The risk of morbidity only increases significantly when the pH is less than 7.0.

### 3.2. Base Deficit (BD)

The base deficit (BD) is indicative of the fetal reserves and represents the number of bases that would neutralize the blood to a pH value ranging between 7.2 and 7.4. The carbonic acid (H_2_CO_3_) −bicarbonate (HCO_3_^−^) buffer is represented by Equation (1) [36].

(1) $$H^{+}+{\mathrm{HCO}}_{3}^{-}↔H_{2}{\mathrm{CO}}_{3}↔{\mathrm{CO}}_{2}+H_{2}O$$

The produced CO_2_ is eliminated through gas exchange in the placenta [37]. The BD is calculated from pCO_2_ and HCO_3_^−^ by using a derived algorithm (Equation (2)) of the Siggard-Andersen chart [1].

(2) $$\mathrm{BD}=-0.9149\cdot \left(0.12\cdot {\mathrm{pCO}}_{2}\cdot {\mathrm{CO}}_{2}\cdot 10^{\mathrm{pH}-6.1}-24+16.21\cdot \left[\mathrm{pH}-7.4\right]\right)$$

The BD helps quantify the metabolic component of an observed change in pH with values above 12.0 mmol/L reflecting severe metabolic acidosis. The pH < 7.00 and BD ≥ 12.0 mmol/L are considered essential criteria for the diagnosis of an acute intrapartum hypoxic event [38] with the BD being used as an outcome measure examining intrapartum interventions [39,40,41]. However, the BD is an artificially calculated parameter and reflects the buffering capacity of the entire body. Different equations are used to calculate the BD and blood gas analyzers use different algorithms for its determination [36]. It is, therefore, recommended that the BD not be used as a sole parameter for the assessment of fetal wellbeing.

### 3.3. Lactate

Lactate, which is the anion that results from the dissociation of lactic acid, is the end product of anaerobic glucose metabolism. In hypoxic conditions, lactate levels in subcutaneous tissues increase before pH decreases. Therefore, it appears to be an earlier marker of evolving metabolic acidosis [42]. Fetal scalp lactate during labor and umbilical artery lactate at delivery have been shown to predict a poor neonatal condition and hypoxic-ischemic encephalopathy better than pH [27]. The introduction of electrochemical devices, which require only 5 µL of blood and enable the measurement of lactate at the bedside, also has the potential to reduce the delays and high failure rate associated with FBS for pH measurement. Several point-of-care sensors have now been validated for intrapartum use with low coefficients of variation. However, it is important to note that actual lactate levels and corresponding thresholds may differ between individual devices. Current devices do not sample continuously [25,26].

Currently recommended clinical thresholds for fetal scalp lactate are outlined in Table 1. Observational data reported by Kruger et al. suggested that the optimal cut-off value to predict moderate or severe hypoxic-ischemic encephalopathy was 6.5 mmol/L [27]. Therefore, a lower threshold for the intervention of 4.8 mmol/L (representing the 75th centile in their high-risk group) was chosen to prevent rather than predict, adverse outcomes. The corresponding value (25th percentile) for fetal scalp pH was 7.21 in the same group. Since this closely aligned with the recommended pH threshold of 7.20, the thresholds above have been widely accepted. There is no difference in neonatal outcomes or operative interventions seen in randomized trials comparing lactate and pH measurement in fetal blood sampled when clinically indicated during labor [35]. However, the rate of sampling and analysis failure appear to be much lower (less than 2%) with point-of-care lactate sensors [43]. For these reasons, lactate is recommended in several national and international guidelines as a more reliable alternative to fetal scalp pH, but its use has not been universally adopted [4,44]. Of note, the combined measurement of pH and lactate does not improve the prediction of adverse outcomes over each method individually [27].

## 4. Clinical Device Requirements for Ideal Fetal Hypoxia Monitoring Sensor

The device requirements, which are shown in Table 2, need to be fulfilled for a new device to be used for fetal bio-sensing during labor.

### 4.1. Accuracy of Measurement

Measurement accuracy and reproducibility is paramount for critical decision-making such as fetal health. The accuracy of the measurement depends on the sensor and the interaction of the sensor with the bodily fluid. The measurement of lactate within the fetal blood and the interstitial fluid (ISF), which is a solution of sugars, salts, fatty acids, amino acids, and cell waste products that constitute part of the extracellular fluid. Using a minimally invasive device can affect the accuracy and stability of the sensors due to biofouling. Biofouling can be caused by factors such as protein adsorption, cellular adhesion, thrombus formation, and more [45]. The dynamic nature of these effects limits the efficacy of regular recalibration to ensure accuracy since it cannot be readily determined whether observed measurement changes are caused by the time-variant properties of these biological films or whether measurement changes are caused by localized changes due to the presence of metabolically active, adhered cells, sensor drift, or genuine physiological change. A practical solution is the selection or modification of materials to prevent these biofouling effects from occurring. Commonly used examples include the application of a thin film of polyethylene glycol (PEG) to prevent protein adsorption [46,47] and the utilization of nitric oxide coatings to eliminate thrombus formation [48] as well as limiting the use of these devices to durations of less than 24 h to minimize these effects.

Another impediment to electrochemical lactate measurement accuracy is the presence of common electroactive interferents such as acetaminophen, ascorbic acid, and uric acid, which adversely affect the specificity of in-vivo electrochemical sensors [49,50]. Recent studies have demonstrated that this interference can be minimized through either the use of compensation electrodes and direct electron transfer [50] or modification of the electrode material [49,51,52].

### 4.2. Frequency of Monitoring

A key parameter that must be selected for lactate detection is measurement frequency. Single FBS samples are both invasive and provide only a single measurement point. More regular measurements require a sampling location where fluids closely reflect the systemic behavior. For example, less invasive measurements obtained from the skin must accept that some lag may be present between systemic lactate levels and those within peripheral blood, interstitial fluid, or sweat. Several studies have demonstrated a good correlation between venous glucose and ISF glucose in human subjects [53,54,55,56]. Some evidence shows that the same is true for lactate [57,58]. For example, in glucose monitoring, there is a lag of 5 to 6 minutes from intravascular injection to interstitial skin fluid presence [59]. In lactate (a smaller molecule), low lag times have been observed in mice [60] but, to our knowledge, this work has not been performed on fetuses. We propose that measuring tissue lactate at 5-minute intervals would provide sufficient notice of lactate level rises, which act as a practical health monitor.

### 4.3. Biocompatibility

The challenge of biocompatibility for sensors to be used during pregnancy is twofold since there is a potential risk to both the mother and the fetus. However, the total duration of sensor use will be less than ~12 h, which reduces the complications of long-term wound healing responses and occurs over several days (e.g., macrophage infiltration [61]). The total duration is known to affect other types of sensors (e.g., continuous glucose monitors [62]).

#### 4.3.1. Risk to the Mother

The risk to the mother can be reduced by using materials and approaches that mirror those used in current devices used in labor such as the FSE or fetal scalp blood sampling kits. Using an applicator device such as the ones used in the FSE should also reduce the risk of soft tissue damage to the mother. Other risks do arise from any potential degradation of the sensor components (e.g., enzyme and substrate), which results from the amniotic fluid, vaginal secretions, and the temperature of the environment.

#### 4.3.2. Risk to the Fetus

Sensors that require the skin to be punctured to reach blood or ISF will need to demonstrate that chemical leaching into the fetus tissue either does not occur or poses no health risk. This is particularly important when considering how the enzyme is immobilized on the sensor surface. For example, devices that make use of Carbon NanoTubes (CNTs) or other materials that risk damaging or irritating tissues [63] will require strong evidence that they are not released within the fetus tissue. Methods to improve biocompatibility (and safety) include employing a biocompatible porous polymer layer to cover the enzyme [64] or locating the enzyme within a hydrogel [65]. Particular attachment chemistries must also be non-reactive within the skin and not invoke any allergic or irritant response. These will require substantial safety testing before any use in a fetus.

### 4.4. Regulatory Device Considerations

There are practical regulatory considerations, which will be affected by device design that must be taken into account. For example, if the device rests on the baby’s scalp and senses sweat, then the device will be classed as non-invasive, which leads to a more straightforward path in clinical use. If a component of the device enters the body and then the risks of infection, tissue reactions, pain and bleeding become greater, then the sensor is minimally perturbing [62]. In this scenario, the regulatory pathway will be substantially more arduous with materials, chemicals, and construction being placed under greater scrutiny. However, this must be balanced against the quality of the signal that can be achieved from the sensor.

## 5. Enzyme-Based Lactate Sensors

Enzyme-based lactate sensors can be categorized based on the immobilization method of the enzymes on the sensor surface and the transduction method used. Immobilization refers to the attachment of recognition elements such as enzymes either to a substrate or within a support material to ensure a response to the analyte. The immobilization method chosen can affect the lifetime, sensitivity, and limit of detection of the sensors [66]. This section describes the various immobilization methods used in the production of electrochemical and optical lactate sensors shown in Table 3 and Table 4.

### 5.1. Enzymes

An enzyme is the most commonly used recognition element for lactate detection. Two different enzymes are used to recognize lactate, which include lactate oxidase (LOx) [65] and lactate dehydrogenase (LDH) [76]. Enzymatic biosensors have some disadvantages that need to be addressed before wider clinical translation. Enzyme performance and, therefore, sensor stability is affected by the thermal and the surrounding chemical environment. Exposure to changes in temperature or pH can affect the performance and stability of these sensors, which should be taken into account when using these materials [94,95]. One recently used method is through the use of genetically-engineered enzymes even though this may lead to further regulatory issues [95]. 

#### 5.1.1. Lactate Oxidase (LOx)

Lactate oxidase (LOx), which is also referred to as lactate mono-oxygenase, promotes the oxidation of lactate to pyruvate in the presence of oxygen forming hydrogen peroxide (H_2_O_2_) [96]. H_2_O_2_ is electrochemically active and can be reduced or oxidized to produce an electrical current that is proportional to the lactate concentration [97]. The reaction does not require a co-factor [85,98]. Compared to LDH, it requires a lower over-potential, which makes it suitable for electrochemical sensing.

(3) $$\mathrm{Lactate}+O_{2}\overset{\mathrm{LOx}}{\to }\mathrm{Pyruvate}+H_{2}O_{2}$$

(4) $$H_{2}O_{2}\to O_{2}+2H^{+}+2e^{-}$$

The oxidation of H_2_O_2_ is preferred as the measurand because the reduction of oxygen is susceptible to oxygen fluctuations in the sample due to other sources [98] requiring simultaneous recording of environmental oxygen to ensure accuracy [96]. The dependence of the enzymatic reaction on oxygen suggests that the effect of oxygen must still be considered when relating the hydrogen peroxide concentration to the lactate concentration especially for in vivo application. Experiments carried out in the rat brain suggest that lactate oxidase-based sensors are reliable for in vivo testing and can tolerate hypoxic conditions. These results suggest that, as long as pO_2_ is not lower than 10 mmHg, the sensor reliability is not adversely affected by the surrounding oxygen [99] but may reduce sensor sensitivity [100]. 

H_2_O_2_ requires a high electrochemical potential to oxidize, which reduces the sensor specificity, due to increases in interference from other substances in the sample such as ascorbic acid, uric acid, or acetaminophen [98]. The interference can be eliminated or reduced by covering the sensing surface with a Nafion^®^ [101] or electro-polymerized thin films [102], which may lead to a decrease in sensitivity.

#### 5.1.2. Lactate Dehydrogenase 

LDH is a protein of great importance for the human body. It is released to break down lactate in damaged tissue or muscles. LDH also promotes the oxidation of lactate to pyruvate. Instead of oxygen, LDH requires the oxidized form of nicotinamide adenine dinucleotide (NAD^+^) for the reaction to occur. NADH, which is the reduced form of NAD^+^, is the outcome of this reaction (Equations (5) and (6)). This can then be oxidized [98]. In amperometric sensors, the oxidation current produced by the oxidation of NADH is directly proportional to the lactate concentration.

(5) $$L\mathrm{actate}+{\mathrm{NAD}}^{+}\overset{\mathrm{LDH}}{\to }\mathrm{Pyruvate}+\mathrm{NADH}+H^{+}$$

(6) $$\mathrm{NADH}\to {\mathrm{NAD}}^{+}+H^{+}+2e^{-}$$

Unlike LOx, LDH is independent of the presence of oxygen [76,103]. Sensing with LDH does, however, have a few disadvantages. LDH is an unstable protein and requires a high over-potential of about 1 V for the oxidation of NADH [104], which can interfere with and reduce the specificity and stability of the sensor [76,105] due to fouling [105]. 

Though the lower over-potential has made lactate oxidase more suitable for the implementation of electrochemical sensors, the fluorescent properties of the co-factor NADH make it attractive for the development of optical lactate sensors [67,74,84,106]. Additional reagents such as luminol are added to achieve more sensitive optical lactate sensors based on LDH [67]. However, luminol is not biocompatible since it causes skin irritation and has a negative impact on the digestive and the respiratory system [107]. Such a reagent is, therefore, not suitable for in vivo applications.

### 5.2. Immobilization Methods 

To detect fetal hypoxia, the sensors need to be in direct contact with human tissue. An immobilization method is, therefore, required, which provides a strong bond between the enzyme and the electrode surface. It also improves stability and sensor lifetime. For patient safety, the method should also prevent leaching. There are several methods of attachment including adsorption, encapsulation, and cross-linking. It is, however, worth noting that current toxicology and biocompatibility data may indicate risks for some of the nanotechnology approaches (e.g., CNTs or Nanoparticles) removing the practicality of clinical translation [108].

#### 5.2.1. Adsorption

Physical adsorption is the non-specific physical attachment of enzymes through Van der Waal forces, hydrogen bonding, or ionic interactions to the sensing surface [109]. This way of immobilization is simple since neither enzymes nor the support needs to be charged. The support also does not require much preparation but the lifetime and reproducibility of sensors manufactured with this method are poor compared to other immobilization methods [66,110] 

Ionic adsorption makes use of the electrostatic interaction between ions. The connection between negatively and positively charged ions is dependent on the surrounding pH, temperature, and ionic strength and forms the foundation for the ion bonding. Since it is a weak bonding, the enzymes do not experience much deformation and the immobilization is easily reversed. This reversal can cause leaching, which makes the sensor less effective and stable [111], and introduces potential biocompatibility problems.

Covalent adsorption is based on covalent bonding in which two atoms share electrons. While this immobilization method is a time-consuming process, this attachment method forms a strong connection between the enzyme and the support material, which makes it more stable and less prone to leaching. However, this immobilization method can lead to reduced enzyme activity due to enzyme deformation, limited enzyme movement, and blocking of active sites [112]. 

#### 5.2.2. Entrapment and Encapsulation

Enzymes can be immobilized by entrapment or encapsulation through the formation of a lattice or matrix, which enclosed them without forming a connection. Due to the minimal modification of the enzyme, activity is maintained and the stability of the sensor is increased. Since there is no connection between the enzyme and the support, the active sites do not get blocked [113] and the effect of interference from other substances is minimized [98]. However, mass transfer can be impaired due to the dependency on the porous network surrounding the enzymes. Typical materials used for entrapment and encapsulation are sol-gel and hydrogel [114]. Sol-gel requires a non-biocompatible precursor and forms alcohol-causing gel-shrinkage during the manufacturing process [112]. 

From Table 3, the sensors using sol-gel or hydrogel technology to entrap/encapsulate the lactate sensing enzymes onto the substrate have the potential to detect lactate concentration as low as 130 nM [76] and 8000 nM [75], respectively. Immobilization through entrapment/encapsulation is mostly used in combination with amperometric transduction. Although good response times (2–7 s) have been achieved [77,78,115], such sensors tend to have a slower response time defined in terms of minutes [65]. This slow response may be due to the diffusion barrier created through the gel around the enzyme, which is also a protective barrier against the surrounding environment.

#### 5.2.3. Cross-Linking

Cross-linking uses a two-step process involving the use of reagents to provide sufficient immobilization. Although this method forms the stable immobilization of the enzyme and does not require additional support such as gels or membranes, it may cause a conformational change, which reduces enzyme performance. This method can be improved by using a support material such as chitosan [112]. 

Table 3 shows that more than half of the listed sensors utilizing cross-linking offer a sufficient detection limit needed for fetal monitoring [49,73,80,81,83,85,91,102,116,117,118]. The response time ranging from 5 s to 180 s for the electrochemical sensors may not be entirely due to the immobilization method but could be the result of different protective layers employed, which does not allow for fast diffusion. However, cross-linking is a common method of immobilizing enzymes. Protocols to achieve this are readily available [72,88,119,120,121]. Out of the various methods of immobilization described, covalent bonding and cross-linking are most likely to provide strong immobilization of the enzyme. 

#### 5.2.4. Immobilization for FBS Lactate Sensors

Table 3 classifies lactate sensors by their method of immobilization. More than half of the lactate sensors surveyed in this literature review are listed as using adsorption to meet the detection limit of 0.001 mM required for FBS [68,69,117,119,122]. Most of those employ amperometry in combination with LOx [77,119]. The sensors with the lowest detection limit also attach nanoparticles to the sensing substrate, which acts to increase the specific surface area of the sensor [68,69]. From this table, an electrochemiluminescence (ECL) sensor also achieved the lowest detection limit [67]. Immobilisation through adsorption seems to meet the desired specifications as well as through the use of fluorescent LDH sensors [74]. 

## 6. Lactate Sensor Transduction Mechanisms

The main transduction methods used for lactate detection are electrochemical [77,85] and optical transduction [65,67] even though a microwave-based transduction method was recently presented [123]. 

### 6.1. Optical Sensing

The measurement of lactate concentration by optical means has been around since 1964 [124] when Broder and Weil measured the blood lactate concentration through the absorption of light. Few sensors still use absorbance as the method of detecting lactate. Fluorescence and chemiluminescence are becoming the two most popular methods due to the lower detection limits possible, which is shown in Table 4. Fluorescence techniques equate the concentration of the analyte with the intensity or lifetime of the light emitted from the analyte upon excitation by light of a specific wavelength. The Parker equation, which is shown in Equation (7), can be used to determine the analyte concentration if light intensity is the measurand where *L*, *I_O_*, *ϕ, ε, C*, *l*, *k* are the luminescence intensity, excitation source intensity, luminescence quantum yield, molar absorbance of the analyte at the excitation wavelength, light path through the analyte solution, and the instrumental constant, respectively [125].

(7) $$L=2.31I_{O}\varphi \varepsilon \left[C\right]lk$$

Lactate dehydrogenase is the most commonly used enzyme in the production of fluorescent lactate sensors since its co-enzyme NADH is known to absorb wavelengths between 330 nm to 360 nm and emit light at wavelengths between 450 nm to 460 nm. The intensity of the light emitted is proportional to the concentration of NADH and the amount of lactate [96]. 

Chemiluminescence transducers depend on the emission of light due to a chemical reaction, which is usually an oxidation reaction caused by oxygen or hydrogen peroxide [112]. Chemiluminescent lactate sensors are primarily achieved by the reaction between hydrogen peroxide produced by the enzymatic reaction and luminol [96]. These sensors are simple to produce and have low detection limits and a wide dynamic range. However, the underlying chemical reaction to generate light requires the use of an alkaline medium (pH 8.5–9) to ensure optimal light efficiency, which is undesirable since enzyme activity requires a pH neutral environment [126]. Various solutions have been proposed for this conundrum such as photomultiplication tubes [127], two-step enzymatic systems [128], and flow injection systems [126,129] at the expense of system simplicity. Furthermore, as previously noted, luminol is not suitable for in vivo applications due to the irritation of the skin and adversely affects the respiratory and digestive systems [107].

Spectroscopic methods have also been utilized to measure lactate concentrations for in-vivo biomedical applications [125,130,131,132,133,134,135,136]. These are commonly used as a non-invasive method of measurement for dermatological analysis where a low-power laser beam in the near infrared range was placed on the skin using an optical fiber or directly scanned over the surface and the resulting reflected light collected and analyzed for the presence of lactate or other analytes. In-vivo studies conducted by Pillotto on anesthetized murine models using Raman spectroscopy were able to identify a change in blood lactate with time, but the limit of detection, sensitivity, and resolution of this method under these conditions was not reported [131]. The investigation of the molecular composition of the stratum corneum, which is the outermost layer of the skin, using confocal Raman spectroscopy was able to detect the presence of lactate but further details were not provided [130]. Other studies using spectroscopic methods have also reported being able to detect changes in the amount of lactate over time or in a given area but do not report whether they can measure the actual concentration [134,136]. A recent study was able to produce maps of the concentration per volume of the constituents of the stratum corneum, such as water, lactate, and lipids. However, no information on the sensor limits and sensitivity were provided even though the risk of heating of the sample and damage to the skin was reported due to the lasers required to achieve these maps [135]. All of these studies demonstrate that the relative concentration of lactate with respect to time or space can be detected. However, the absolute concentration cannot be detected, which limits the applicability of these methods for fetal monitoring.

Although optical sensing holds several advantages over electrochemical sensing such as immunity to electromagnetic interference, electrical isolation from the patient, and ease of miniaturization, comparatively few researchers have explored optical detection as an alternative method for determining lactate concentration for in vivo applications. Reasons for this may be due to the fact that the response of optical sensors can vary from person to person due to skin pigmentation and epidermal thickness as well as the scattering of light in tissue and the risk of photo-bleaching of the skin, which have impacted the development of continuous optical glucose monitoring [62]. 

### 6.2. Electrochemical Sensing

The majority of the lactate sensors utilize amperometry, which is an electrochemical method for the analysis of electrochemical cell reactions through the detection of changes in electrical current. A step in voltage potential, which ranges from −0.2 V to 0.7 V, is applied to a working electrode with respect to a reference electrode [76,99,115]. Potential electrolysis (reduction or oxidation) takes place, which is measured as a change in electrical current over time. The electrical current response is proportional to the concentration of the analyte submitted to the chemical reaction [137]. For planar electrodes, this response is described by the Cottrell equation shown in Equation (8).

(8) $$i=\frac{nFAD^{0.5}C}{{\left(\pi t\right)}^{0.5}}$$

where *n* is the number of electrons, *F* is the Faraday constant, *A* is the electrochemical surface area (cm^2^), *D* is the diffusion coefficient (cm^2^ s^−1^), *C* is the concentration of the electroactive species (mol cm^3^), and *t* is the time in seconds [138]. Amperometric lactate sensors measure the current flow generated by electrons, which have been produced either through the breakdown of H_2_O_2_ or NADH. These intermediates originate from LOx and LDH, respectively, which is explained above. The electrical current measured upon the application of the overpotential is a function of the concentration of the lactate in the solution. 

The amperometric lactate sensors are summarized in Table 4. All sensors listed achieve a detection limit of 3 pM to 600 nM [68,116]. However, only two of those react within 1 to 6 s [68,73]. The working electrodes have been modified using different materials such as the multi-walled carbon nanotube (MWCNT) [69,77,139,140], chitosan [141], nanowires [68], and nanoparticles [76,119]. The functionalization before the enzyme immobilization is demonstrated to be one of the more important fabrication steps of a lactate sensor. The sensors with the quickest reaction time utilizes some form of nanomaterial since this is thought to generate an increased electrochemical surface area for attaching the enzyme and for recognizing the lactate. Wei et al. have developed an amperometric lactate sensor based on a platinum-chitosan electrode functionalized with lactate oxidase, which achieves a detection limit of 50 nM within 1 s over a period of 24 h [141]. Chitosan is made from the chitin shell of shrimps and is biocompatible and non-toxic. Windmiller et al. used carbon paste in microneedles in combination with amperometric transduction [49]. Their research found negligible interference of other substances. However, their operational stability was only tested for two hours. Ma et al. developed a lactate sensor using a high electron mobility transistor (HEMT) and zinc oxide (ZnO) nanowires [68]. In this HEMT based sensor, the drain current represents the lactate concentration in the sample. Furthermore, the presented sensor does not require a reference electrode, which makes it possible to minimize the required size of the gate. The gate is fabricated from aluminum gallium arsenide (AlGaAs), which has high chemical stability and good electron transport properties. However, toxic effects have been reported for AlGaAs, which makes it unsuitable for medical application with regard to potential direct contact with the patient [142]. 

## 7. Conclusions

Current research studies seem to indicate that electrochemical sensing may be the preferred modality of lactate sensors whereby either lactate oxidase or lactate dehydrogenase are immobilized onto the working electrode. Both enzymes have been shown to successfully provide a basis for lactate sensing. However, a significant proportion of published research focuses on the use of lactate oxidase as lactate dehydrogenase, which is a less stable protein and requires a larger over-potential.

Covalent bonding and cross-linking have been identified as the most suitable means for immobilizing the enzymes to the electrode surface. These methods have been found to prevent leaching of the enzyme, which is important when in contact with human tissue. Encapsulation requires an additional gel that is not compatible with minimally invasive methods of delivery such as microneedles.

The study of the available literature has shown that amperometry is the most common transduction method for lactate sensing. Other means of transduction have been researched but are not as advanced in their development. The materials used for the working electrode of lactate sensors include gold, platinum, carbon, and glass, which can be further modified with nanomaterials to improve sensitivity. Alternative methods of improving sensor performance will need to be investigated. Since nanomaterials can potentially leach and be toxic, they cannot be utilized for the development of a fetal sensor. 

Finally, the final sensor application must ensure that clinical decisions can be made from the data obtained from the sensor, which means that minimal lag time and clear data are paramount. The accuracy and stability of the sensors, which is vital for clinical applications, may be enhanced through the use of multi-modal sensing including the integration of pH and lactate sensing on the same platform to compensate for changes in sensor performance due to localized environmental changes. Achieving this in a small, easy-to-use sensor that can withstand the birthing environment has great potential to reduce acidosis during birth.

## Figures and Tables

**Table 1:** Interpretation of fetal scalp blood sampling pH and lactate values.

Measurands		Interpretation
pH	Lactate	
≥7.25	≤4.1 mmol/L	Normal
7.21–7.24	4.2–4.8 mmol/L	Borderline
≤7.20	≥4.9 mmol/L	Abnormal

**Table 2:** Desirable device requirement for detection of fetal hypoxia.

Parameter	Functionality
**External environment**	The device should maintain accuracy and functionality when exposed to vaginal secretions, which have a pH of 3.8–4.5 amniotic fluid, which has a pH of 7–7.5 a temperature of 35 °C to 42 °C (maternal environment—external connections and fetal scalp pH—sampling device) movement during contractions and fetus movement down the birth canal
**Biological fluid sampled**	The device has the potential to sample fetal scalp interstitial fluid and/or fetal blood
**Device dimensions and specifications**	The probe should be able to be affixed to the fetal scalp through a cervix, which is 1 cm or more dilated. The probe should be no more invasive than an existing FSE. The probe should be easy to attach and remove from the fetal head while remaining immobilized against the scalp during contractions or fetal movements. The device should be compatible and not interfere with existing external methods of fetal monitoring (e.g., external CTG). No separate fetal blood gas/lactate analyzer was required. The device should be capable of continuous sampling (<5 min intervals) and measurement of the measurand (s) for a minimum of 12 h. The material must not degrade during sterilization.
**Biocompatibility**	The materials used in construction should be biocompatible. Any chemicals used for sensing must be affixed in a way that ensures that they do not separate from the sensor surface.

**Table 3:** Summary of various lactate sensors from literature arranged according to the attachment method of the enzyme.

Substrate	Enzyme	Attachment Method	Material	Transduction	Application	Response Time [s]	Sensitivity	LoD [pM]	Range of Detection [pM]	Reference
Polymer	LOx	E (Ent)	Carbon paste	AM	Test solution	--	--	4.20 × 10^8^	4.20 × 10^8^–8.00 × 10^8^	[49]
Pt	LOx	C	Pt	AM	Blood and EISF	120	0.2252 µA mM^−1^	4.44 × 10^6^	3 to 13 mg dL^−1^	[58]
NA	LOx	E (HG)	HG	P	Test solution	912	171.52 μA mM^−1^	4.44 × 10^7^	--	[65]
Pt	LDH	A	CNTs	ECL	Test solution, sweat	--	--	8.90	8.90–8.90 ×10^6^	[67]
AlGaAs	LOx	A	In-doped ZnO NW	AM	Test solution	10	--	3.00	3.00–3.00 ×10^9^	[68]
GC	LDH	A	NPs/MWCNT	AM	Serum	--	7.67 μA mM^−1^	5.00 × 10^6^	5.00 × 10^7^–5.00 × 10^8^	[69]
GC	LOx	A	Carbon	ECL	Human serum	--	--	2.00 × 10^6^	2.00 × 10^6^–2.00 × 10^8^	[70]
GC	LOx	A	CNT	AM	Test solution	2	40.00 µA mM^–1^ cm^–2^	4.10 × 10^6^	1.40 × 10^7^–3.25 × 10^8^	[71]
Carbon	LOx	A	Pt-NPs/GCNF-SPCEs	AM	Food samples	--	41.30 ± 546 μA mM^−1^ cm^−2^	6.90 × 10^6^	1.00 × 10^7^–3.25 × 10^8^	[72]
Pt	LOx	A	NPs	AM	Test solution	6	0.0002 µA mM^−1^	1.00 × 10^2^	5.00 × 10^8^–1.55 × 10^10^	[73]
OF	LDH	A	--	F	Test solution, single cell	1	--	2.00 × 10^7^	6.00 × 10^7^–1.00 × 10^9^	[74]
Glass	LOx	E (HG)	NA	AM	Test solution	20	0.0662 µA mM^−1^	8.00 × 10^7^	8.00 × 10^7^–9.00 × 10^10^	[75]
Graphene	LDH	E (SG)	Au NPs	AM	Artificial serum	8	154 µA mM^−1^ cm^−2^	1.30 × 10^5^	1.00 × 10^7^–5.00 × 10^9^	[76]
GC	LOx	E (SG)	Pt NP. MWCNTs	AM	Whole blood	5	6.36 μA mM^−1^	3.00 × 10^8^	2.00 × 10^8^–2.00 × 10^9^	[77]
Au	LOx	E (Ent)	Chitosan/CNT	AM	Test solution	7	19.7 μA mM^−1^ cm^−2^	5.00 × 10^6^	--	[78]
Graphite	LOx	E (Ent)	Chitosan/CNT	AM	Test solution, cell culture	--	3.417 µA mM^−1^	2.26 × 10^7^	3.04 × 10^7^–2.44 × 10^8^	[79]
GC	LOx	E (SG)	Polymer	AM	--	--	1.02 μA mM^−1^	5.00 × 10^7^	1.00 × 10^8^–9.00 × 10^9^	[80]
Polyimide	LOx	E (HG)	Pt	AM	--	30	0.005 µA mM^−1^ mm^−2^	--	--	[81]
Plastic	LOx	E (HG)	Glass	AM	Test solution, dialysate	144	0.00027 µA mM^−1^	--	0–1.5 × 10^10^	[82]
Pt	LOx	E (SG)	SiOx	AM	Test solution	--	180 µA mM^−1^ cm^−2^	--	2.00 × 10^9^–8.00 × 10^9^	[83]
Glass	LDH	E (SG)	Si	ECL	Test solution	--	--	--	--	[84]
Glass ceramic	LOx	C	Au thin film	AM	Test solution, wine	15	37.1 μA mM^−1^ cm^−2^	5.00 × 10^6^	5×10^6^–1 × 10^9^	[85]
Glass	LOx	C	Al Au	OFET	Test solution	--	--	6.60 × 10^4^	0–1 × 10^12^	[86]
Glass	LOx	C	ZnO NR	AM	Test solution	10	41.33 ± 1.58 mV/decade	1.00 × 10^6^	1 × 10^8^–1 × 10^12^	[87]
Pt	LOx	C	HG mucin/albumin	AM	Blood	90	0.537 µA mM^−1^	8.00 × 10^5^	2 × 10^6^–1 × 10^9^	[88]
Glass	LOx	C	Carbon film	AM	Test solution/rat brain	--	--	2.30 × 10^6^	5 × 10^6^–5 × 10^9^	[89]
Pt	LOx	C	Monomer	AM	Food samples	60	--	8.00 × 10^6^	8 × 10^6^–1 × 10^9^	[90]
PVC	LOx	C	PB nanocubes	CV	Test solution	5	6.379 μA mM^−1^ cm^−2^	1.00 × 10^7^	1 × 10^7^–5 × 10^8^	[91]
Pt	LOx	C	SPEES/PES	AM	Rats	180	0.001 µA mM^−1^	--	0–7 × 10^9^	[92]

A = Adsorption, AlGaAs = Aluminium gallium arsenide, AM = Amperometric, C = Cross-linking, CNT = Carbon Nanotube, CV = cyclic voltammetry, E = Encapsulation, ECL = electrochemiluminescent, Ent = Entrapment, EISF = Extracellular Interstitial Fluid, F = Fluorescence, GC = Glassy carbon, HG = Hydrogel, LDH = Lactate dehydrogenase, Lox = Lactate oxidase, NP = Nanoparticle, NW = Nanowire, MWCNT = Multiwalled Carbon Nanotube, OF = Optical fibre, OFET = organic field effect transistor, P = Phosphorescence, PB = Lead, Pt = Platinum, SG = Solgel, Si = Silica, SiOx = Siloxane, SPEES/PES = Sulphonated polyether ether sulphone_polyether sulphone, ZnO = Zinc Oxide.

**Table 4:** Lactate sensors based on optical detection.

Substrate	Enzyme	Material	Transduction	Application	Response Time [s]	Sensitivity	LOD [pM]	Range of Detection [pM]	Reference
None	LOx	HG	Phosphorescent	Test solution	912	171.52 μA mM^−1^	4.44 × 10^7^	--	[65]
Pt	LDH	CNTs	ECL	Test solution, sweat	--	--	8.90	8.90–8.90 × 10^6^	[67]
GC	LOx	Carbon	ECL	Human serum	--	--	2.00 × 10^6^	2.00 × 10^6^–2.00 × 10^3^	[70]
OF	LDH	Al coating	ECL	Test solution, single cell	1	--	2.00 × 10^7^	6.00 × 10^7^–1.00 × 10^9^	[74]
Glass	LDH	Si	Fluorescence	Test solution	--	--	--	--	[84]
OF	LOx	PDMS	Colorimetric	Test solution	130	--	5.20 × 10^8^	--	[93]

Pt = Platinum, GC = glassy carbon, OF = Optical fiber, LDH = Lactate dehydrogenase, Lox = Lactate oxidase, CNTs=Carbon nanotubes, Al = Aluminium, PDMS = Polydimethylsiloxane, HG = Hydrogel, Si = Silica, ECL = Electrochemiluminescene, CCD = Charged-coupled device, -- = Not available.
